# Expression of Protein Kinase C Isoforms in Pancreatic Islets and Liver of Male Goto-Kakizaki Rats, a Model of Type 2 Diabetes

**DOI:** 10.1371/journal.pone.0135781

**Published:** 2015-09-23

**Authors:** Mohammed Seed Ahmed, Julien Pelletier, Hannes Leumann, Harvest F. Gu, Claes-Göran Östenson

**Affiliations:** 1 Department of Molecular Medicine and Surgery, Karolinska Institutet, Karolinska University Hospital, Solna, Stockholm, Sweden; 2 Department of Physiology, Faculty of Medicine, University of Khartoum, Khartoum, Sudan; Univeristy of California Riverside, UNITED STATES

## Abstract

Protein kinase C (PKC) is a family of protein kinases controlling protein phosphorylation and playing important roles in the regulation of metabolism. We have investigated expression levels of PKC isoforms in pancreatic islets and liver of diabetic Goto-Kakizaki (GK) rats with and without insulin treatment to evaluate their association with glucose homeostasis. mRNA and protein expression levels of PKC isoforms were assessed in pancreatic islets and liver of Wistar rats and GK rats with or without insulin treatment. PKCα and PKCζ mRNA expressions were down-regulated in islets of GK compared with Wistar rats. PKCα and phosphorylated PKCα (p-PKCα) protein expressions were decreased in islets of GK compared with insulin-treated GK and Wistar rats. PKCζ protein expression in islets was reduced in GK and insulin-treated GK compared with Wistar rats, but p-PKCζ was decreased only in GK rats. Islet PKCε mRNA and protein expressions were lower in GK compared with insulin-treated GK and Wistar rats. In liver, PKCδ and PKCζ mRNA expressions were decreased in both GK and insulin-treated GK compared with Wistar rats. Hepatic PKCζ protein expression was diminished in both GK rats with and without insulin treatment compared with Wistar rats. Hepatic PKCε mRNA expression was down-regulated in insulin-treated GK compared with GK and Wistar rats. PKCα, PKCε, and p-PKCζ expressions were secondary to hyperglycaemia in GK rat islets. Hepatic PKCδ and PKCζ mRNA expressions were primarily linked to hyperglycaemia. Additionally, hepatic PKCε mRNA expression could be under control of insulin.

## Introduction

Type 2 Diabetes (T2D) is a heterogeneous disorder characterized by chronic hyperglycaemia. The aetiological heterogeneity is suggested by genetic inheritance and its interplay with environmental factors. The most important pathophysiological features are impaired insulin secretion and decreased insulin sensitivity (insulin resistance), the latter related to the liver and extrahepatic tissues, mainly skeletal muscle and adipose tissue [1–4].

Goto-Kakizaki (GK) rat is an animal model of T2D derived from repeated selective breeding of Wistar rats with slight impairment of glucose tolerance leading to a non-obese rat strain, which develops diabetes in early life and is characterized by markedly reduced glucose-stimulated insulin secretion (GSIS). The GK rat has been commonly used in genetic and functional analyses of pancreatic hormones and glucose metabolism in relation to T2D. The GK rats of the Stockholm rat colony are descendants from the progenitors of the F40 generation of the original colony [5–9].

In pancreatic islets, skeletal muscle, adipose tissue as well as liver, protein kinase C (PKC) seems to have important roles in mechanisms regulating glucose homeostasis [10–18]. PKC is a family of protein kinase enzymes that are involved in controlling the functions of other proteins through the phosphorylation of hydroxyl groups of serine and threonine amino acid residues on those proteins. PKC isoforms play important regulatory roles in a great number of cellular processes, ranging from fundamental activities such as cell growth and differentiation, proliferation, gene expression, signal transduction, secretion and exocytosis, and smooth muscle contraction, to more organismal functions like memory [19,20]. According to their enzymatic properties, PKC isoforms have been grouped into smaller subfamilies: conventional PKCs (cPKCs), which consist of α, β1, β2, and γ isoforms, are activated by phosphatidylserine (PS), Ca^2+^, and diacylglycerol (DAG); novel PKCs (nPKCs), which comprise δ, ε, θ, and η isoforms, are activated by PS and DAG but not Ca^2+^; atypical PKCs (aPKCs), encompassing ζ and ι\λ isoforms, require neither Ca^2+^ nor DAG for activation; and PKC-related kinases (PRKs), which contain at least three members (PRKs 1–3), are insensitive to Ca^+2^ and DAG [21,22].

In the GK rat pancreas, diminished expression of at least four different PKC isoenzymes, namely PKCα, PKCε, PKCθ, and PKCζ, has been demonstrated by immunohistochemical staining [23]. Recently, PKCδ was suggested to be implicated in insulin sensitivity [24]. In addition to its previously suggested role in treatment of insulin resistance, inhibition of PKCε has been proposed to act as a positive regulator of insulin availability [25].

In the present study, we have investigated mRNA and protein expression levels of PKC isoforms in pancreatic islets and liver of GK rats to assess different levels of functional regulation. To evaluate their association with glucose homeostasis, we treated GK rats with insulin to normalize their plasma glucose levels and, thus, find out whether the altered gene expression, if detected, in pancreatic islets and liver of GK rats is a primary defect or secondary to hyperglycaemia.

## Materials and Methods

### Ethics statement

This study was approved by Stockholms Norra Djurförsöksetiska nämnd (Permit Number: N 224/10).

### Animals

All animal experiments were approved by the regional ethics committees.

Twenty male GK rats, at the age of 2.5-month, were obtained from our colony at Karolinska University Hospital (Stockholm, Sweden), and 10 age-matched male Wistar rats from a local breeder (B&K Universal, Sollentuna, Sweden) were used as controls. Half of the GK rats were implanted with sustained release insulin chips containing 26 ± 2 mg microrecrystallized bovine insulin palmitic acid (LinShin Inc, Ontario, Canada) for 14 days. All animals were kept at 22°C on 12/12-hour light/dark cycle with food and water available *ad libitum*. Plasma glucose levels and body weights were serially measured during the 14-day period. In addition, blood samples collected were placed into ice-cold heparinised tubes, plasma was immediately separated by centrifugation (8000xg, 10 min, 4C) and plasma insulin was quantified using a radioimmunoassay [26]. Samples of pancreas and liver were collected when the rats were sacrificed on the 15^th^ day. Tissue samples i.e liver and isolated pancreatic islets (see below) for mRNA expression studies were stored with RNAlater (Ambion, Austin, USA) at -20°C and those for protein expression studies were cooled in liquid nitrogen and stored at -80°C.

### Isolation and incubation of rat islets

Isolation of rat islets was performed as previously described [27]. Briefly, pancreata were removed after retrograde injection in the pancreatic duct of 24 mg or 9 mg collagenase in 10 ml Hank’s solution (SVA, Uppsala, Sweden) in GK or Wistar rats, respectively. The pancreata were then incubated at 37°C for 24 min and homogenized by suction using a needle into a syringe. Histopaque gradient by mixing the pancreata with 5 ml 1119 and 5 ml 1077 Histopaque solutions (Sigma-Aldrich, Ayrshire, UK) was made after three washing steps. Following centrifugation of the homogenate at 2000 rpm for 20 min, islets were collected from the border of the upper two layers and transferred to Hank’s medium.

### Insulin secretion from pancreatic islets

The isolated islets were incubated to study glucose-stimulated insulin secretion (GSIS). After a pre-incubation period of 30 min at 37°C in Krebs-Ringer bicarbonate (KRB) buffer solution, supplemented with 2 mg/ml of bovine serum albumin, 10 mmol/l HEPES and 3.3 mmol/l glucose, pH 7.4, batches of three islets were incubated for 60 min at 37°C in 200 μl of KRB as above and with 16.7 mmol/l glucose. After incubations, aliquots of the media were taken for radioimmunoassay of insulin [26].

### RNA extraction and real-time RT-PCR

Total cellular RNA was extracted from rat samples using RNeasy mini kits, following the manufacturer’s protocol for tissues (Qiagen, Hilden, Germany). To minimize the risk of RNA degradation, the samples were kept on ice when not performing the extraction, and all working surfaces and tools were cleaned with RNase Away solution (Sigma, Buchs, Switzerland) prior to use. Reverse-transcription (RT) for cDNA from mRNA samples was accomplished using QuantiTect reverse transcription kits (Qiagen, Hilden, Germany).

Real-time RT-PCR was performed with specific TaqMan gene expression assays (Life Technologies, Grand Island, USA) for PKCα, PKCδ, PKCε, PKCζ, and glyceraldehyde-3-phosphate dehydrogenase (GAPDH) genes in rat (Applied Biosystems, Foster City, USA) and with an ABI 7300 real-time PCR system (Applied Biosystems). The ID numbers of TaqMan gene expression assays for studying PKCα, PKCδ, PKCε, PKCζ, and GAPDH genes in rat were Rn01496145_m1, Rn00440891_m1, Rn01785893, Rn00574583_m1, and Rn01476455_m1, respectively. The probes of TaqMan gene expression assays were labelled with carboxyfluorescein (FAM) as a reporter dye and tetramethylrhodamine (TAMRA) as a quencher dye. Amplifications were performed using the 5′-nuclease TaqMan method with a two-step PCR protocol (95°C for 10 min, followed by 40 cycles of 95°C for 15 s and 60°C for 1 min) in an ABI 7300 real-time PCR system (Applied Biosystems). Experiments were replicated at least twice.

### Western blot, antibodies, and reagents

Proteins were extracted from isolated pancreatic islets and frozen liver using a RIPA lysis buffer containing 1 mg/ml phenylmethylsulfonyl fluoride (PMSF), 1 mmol/l Na3VO4, 1 mmol/l NaF (Sigma-Aldrich), 1x protease inhibitors cocktail (Roche Diagnostics), 1x phosphatase inhibitors cocktail (Roche Diagnostics). Liver tissue lysis was performed by a tissue homogenizer (PT-2000, Polytron, Kinematica AG) and the islets were homogenized by 3 x 10 s pulses of sonication (Sonifier 250, Branson). Denaturizing samples were separated on SDS-PAGE and blotted onto nitrocellulose membranes (Sigma-Aldrich). After blocking with 5% fat-free milk, membranes were probed for PKCα, PKCδ, PKCε, PKCζ, phosphorylated PKC (p-PKC), i.e. p-PKCα, p-PKCδ, p-PKCε, p-PKCζ, and GAPDH protein detection using appropriate antibodies: anti-PKCα (sc-208, Santa Cruz Biotechnology), anti-PKCδ (9616, Cell Signaling), anti-PKCε (2683, Cell Signaling), anti-PKCζ (9368, Cell Signaling), anti-p-PKCα (sc-12356, Santa Cruz Biotechnology), anti-p-PKCδ (9374, Cell Signaling), anti-p-PKCε (sc-12355, Santa Cruz Biotechnology), and anti-p-PKCζ (sc-271962, Santa Cruz Biotechnology). Appropriate horseradish peroxidase (HRP)-conjugated secondary antibody was used for detection: HRP-conjugated anti-rabbit (7074, Cell Signaling) and HRP-conjugated anti-mouse (sc-2005, Santa Cruz Biotechnology). Proteins were visualized using an enhanced chemiluminescence procedure (34080, SuperSignal West Pico Cheluminescent Substrate, Thermo Scientific) or (p90720, Immoblion Western, Chemulinescent HRP substrate, Millipore). Quantification was carried out using Luminescent Image Analyzer (Image Reader LAS-100 Pro v1.0, Fujifilm) and ImageJ software (v1.47b, National Institute of Health).

### Statistical analysis

Data were analyzed using GraphPad Prism (v6.0, GraphPad Software). To normalize the skewed distribution, natural logarithmic transformation was applied when needed. The differences in continuous variables between the groups of Wistar rats and GK rats with and without insulin treatment were evaluated with one-way ANOVA followed by Tukey post-hoc test for the mRNA expression analysis, or by Newman-Keuls post-hoc test for the Western blot analysis. Data were given as means ± SE or geometric means (95% confidence interval (CI)). P-value of 0.05 or less was considered significant.

## Results

**Fig 1:**
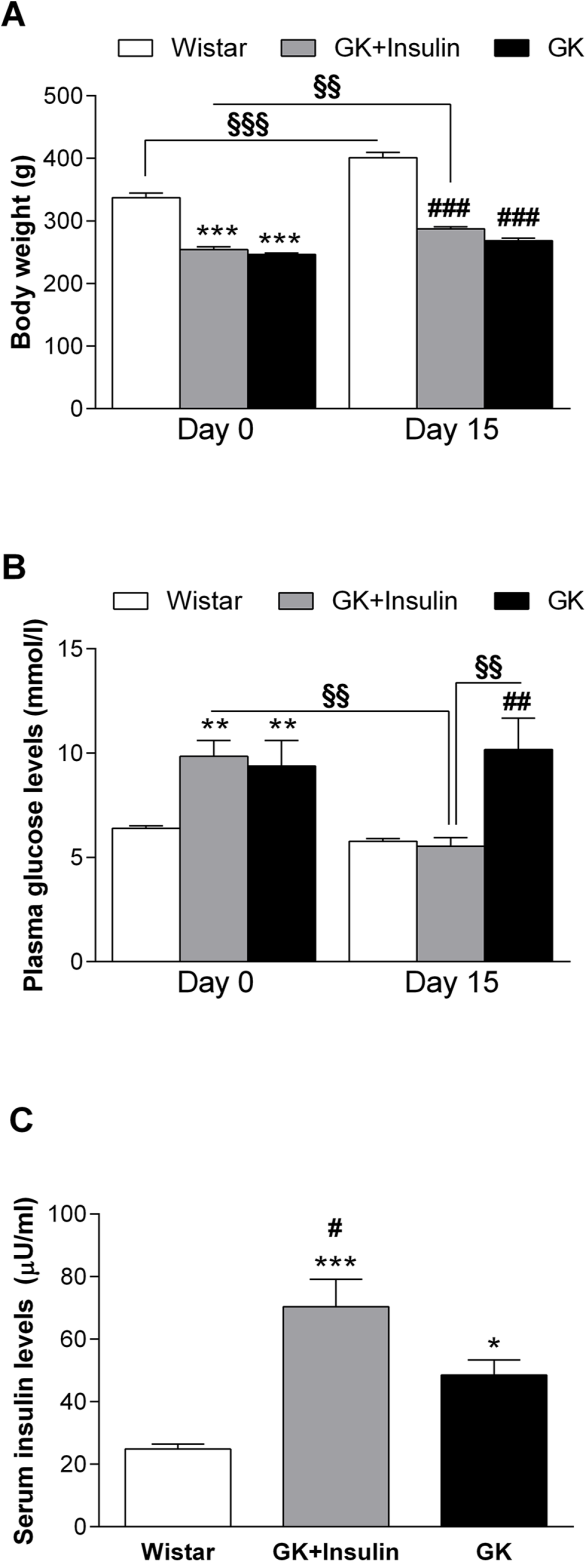
Characteristics of diabetic GK and control Wistar rats. Before and after the 14-day treatment period, the body weights (A) and plasma glucose levels (B) were assessed in Wistar, insulin-treated GK, and non-treated GK rats. After the 14-day treatment period, the serum insulin levels (C) were assessed in the three groups of rats. Data are means ± SE (n = 10). *p < 0.05, **p < 0.01, ***p < 0.001 vs. Wistar rats; ^#^p < 0.05, ^##^p < 0.01, ^###^p < 0.001 vs. GK rats. ^§§^p < 0.01, ^§§§^p < 0.001.

At day 0, the body weights in GK rats (246 ± 3) and GK rats with insulin treatment (255 ± 5 g) were lower compared with Wistar rats (337 ± 7 g, p < 0.001 for both) (Fig 1A). After the 14-day treatment period, the body weights increased in Wistar rats (401 ± 9 g, p < 0.001) and GK rats with insulin treatment (287 ± 3 g, p < 0.01) compared with the respective control at day 0 (337 ± 7 g and 255 ± 5 g, respectively). Nevertheless, after the treatment period, the body weights remained lower in GK rats (268 ± 4 g) and GK rats with insulin treatment (287 ± 3 g) compared with Wistar rats (401 ± 9 g, p < 0.001 for both).

Plasma glucose levels at day 0 in GK rats (9.4 ± 1.2 mmol/l) and GK rats with insulin treatment (9.8 ± 0.8 mmol/l) were higher compared with Wistar rats (6.4 ± 0.1 mmol/l, p < 0.01 for both) (Fig 1B). However at day 15, plasma glucose levels in GK rats (10.2 ± 1.5 mmol/l, p < 0.01) were higher compared with Wistar rats (5.8 ± 0.1 mmol/l) and GK rats with insulin treatment (5.5 ± 0.4 mmol/l) (Fig 1B), while serum insulin levels in insulin-treated GK rats (70.4 ± 8.7 μU/ml) were higher compared with Wistar rats (24.9 ± 1.5, p < 0.001), as well as GK rats without insulin treatment (48.5 ± 4.9 μU/ml, p < 0.05) (Fig 1C). Moreover, serum insulin was higher in GK rats compared with Wistar rats (p < 0.05) (Fig 1C).

Insulin secretion from isolated pancreatic islets at 16.7 mmol/l glucose was less in GK (11.4 ± 2.7 μU/islet/h, p < 0.001) and insulin-treated GK (26.5 ± 1.9 μU/islet/h, p < 0.01) compared with Wistar rats (79.2 ± 12.2 μU/islet/h). Furthermore, insulin secretion was higher in insulin-treated GK rats compared with non-treated GK rats (p < 0.05).

**Fig 2:**
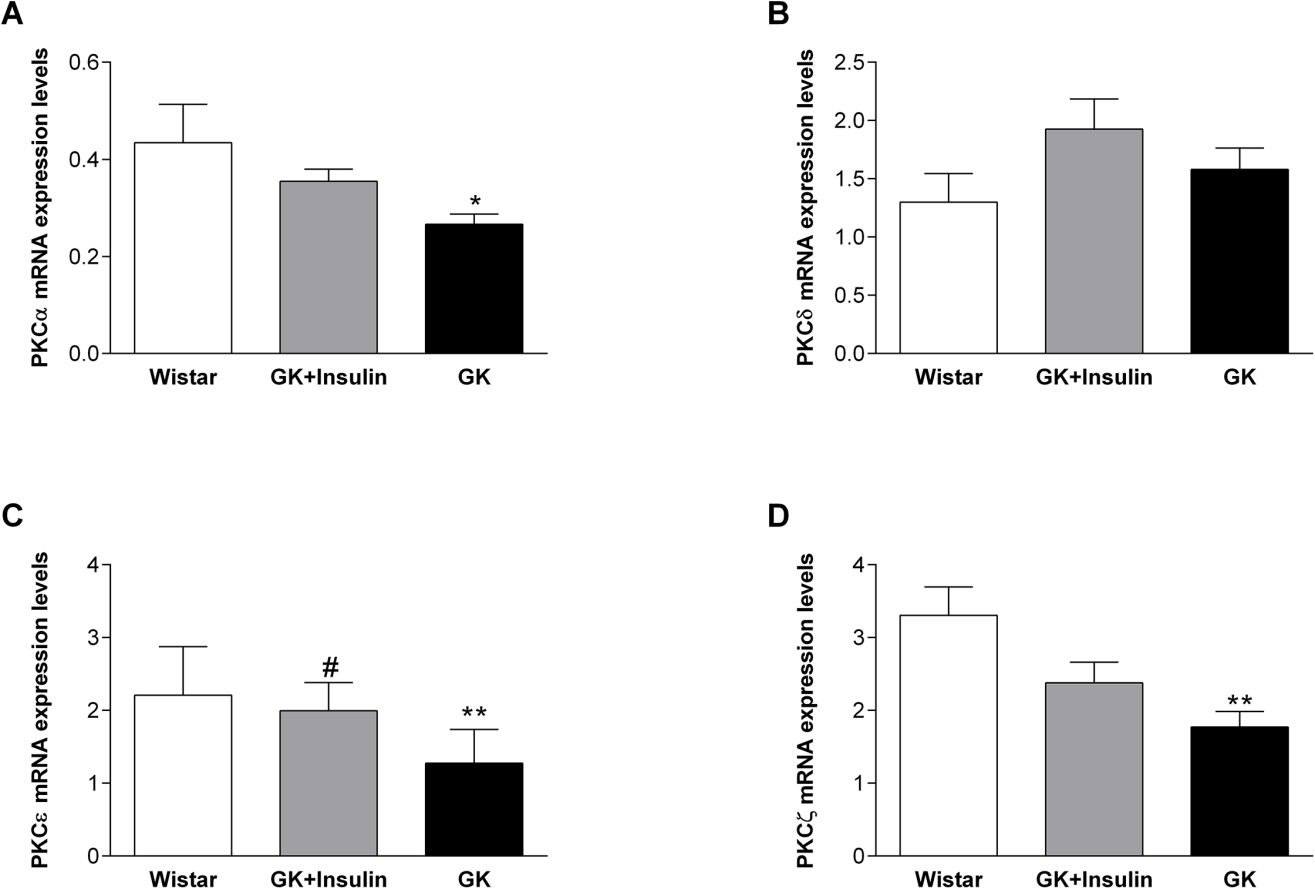
mRNA expressions of PKC isoforms in pancreatic islets. mRNA expression levels of PKCα (A), PKCδ (B), PKCε (C), and PKCζ (D) in pancreatic islets of Wistar, insulin-treated GK, and non-treated GK rats are shown. Data are means ± SE (n = 10) for all PKC isoforms, except for PKCε, for which data have been shown as geometric means (95% Cl) (n = 10). *p < 0.05, **p < 0.01 vs. Wistar rats; ^#^p < 0.05 vs. GK rats.

**Fig 3:**
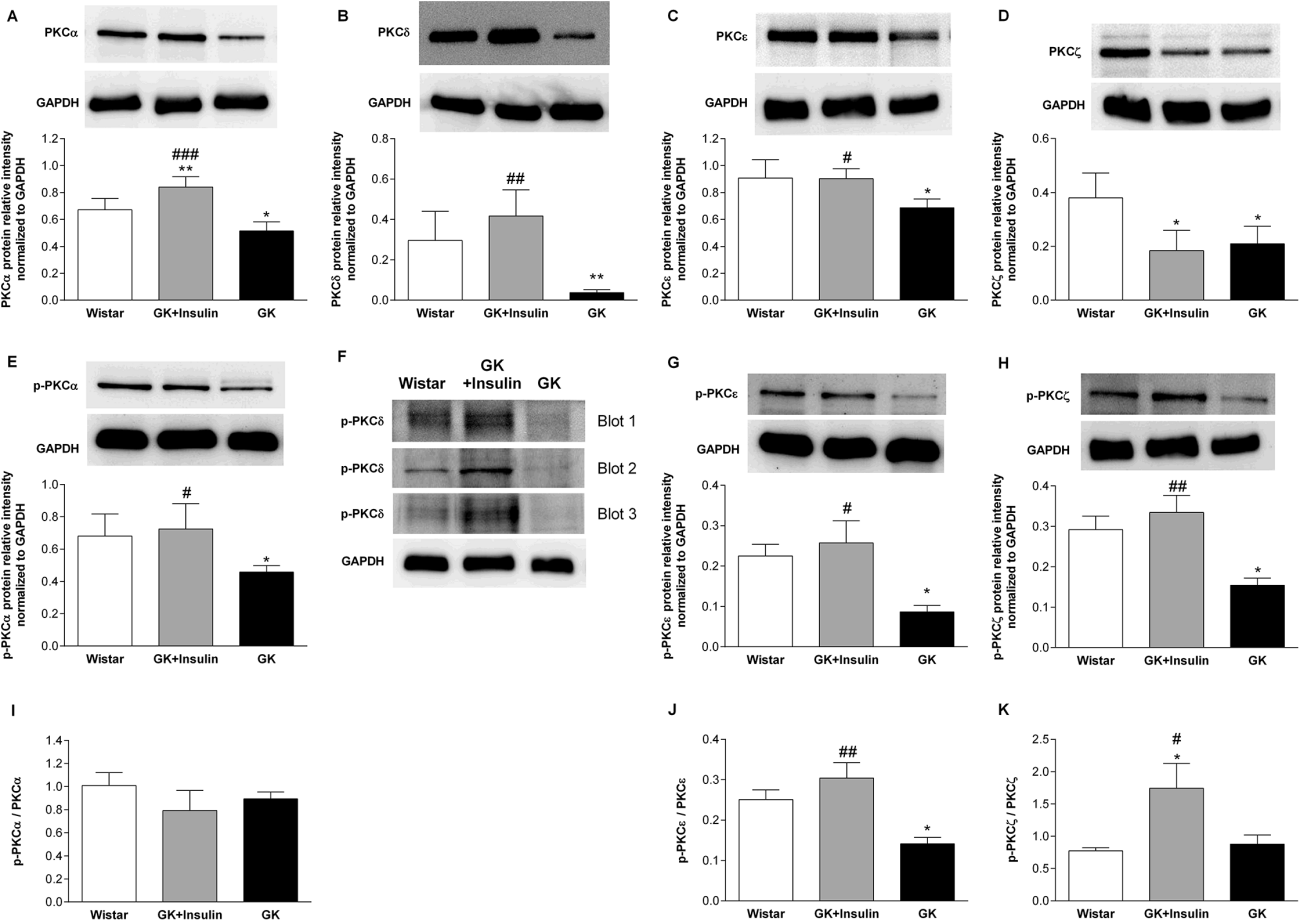
Protein expressions of PKC isoforms in pancreatic islets. Representative immunoblots and densitometric analyses of PKCα (A), PKCδ (B), PKCε (C), PKCζ (D), p-PKCα (E), p-PKCε (G), and p-PKCζ (H) protein expressions in pancreatic islets of Wistar, insulin-treated GK, and non-treated GK rats are presented. The relative expression of p-PKCδ (F) was too low for densitometric analysis, thus p-PKCδ expression is shown with three different representative Western blots. Calculation of the ratio of phosphorylated protein to total protein allowed for assessing the sensitivity of proteins in pancreatic islets from the three groups of rats (I-K). Data means ± SE (n = 4). *p < 0.05, **p < 0.01 vs. Wistar rats; ^#^p < 0.05, ^##^p < 0.01, ^###^p < 0.001 vs. GK rats.

Islet PKCα and PKCζ mRNA expressions were down-regulated in GK compared with Wistar rats (p < 0.05 and p < 0.01, respectively), while their expressions in insulin-treated GK rats were intermediate between GK and Wistar rats (Fig 2A and 2D). PKCα and p-PKCα protein expressions were reduced in islets of GK compared with insulin-treated GK (p < 0.001 and p < 0.05, respectively) and Wistar rats (p < 0.05) (Fig 3A and 3E). Moreover, PKCα protein expression was increased in islets of insulin-treated GK compared with Wistar rats (p < 0.01). PKCζ protein expression in islets was decreased in both GK and insulin-treated GK compared with Wistar rats (p < 0.05), but p-PKCζ was diminished only in GK compared with insulin-treated GK (p < 0.01) and Wistar rats (p < 0.05) (Fig 3D and 3H). Islet PKCε mRNA expression was lower in GK compared with insulin-treated GK (p < 0.05) and Wistar rats (p < 0.01) (Fig 2C). At the protein level, PKCε and p-PKCε expressions were decreased in GK compared with insulin-treated GK (p < 0.05) and Wistar rats (p < 0.05) (Fig 3C and 3G). As to islet PKCδ mRNA, no variation was noticed between the three groups of rats (Fig 2B). However, PKCδ protein expression was reduced in islets of GK compared with insulin-treated GK (p < 0.01) and Wistar rats (p < 0.01) (Fig 3B). By the same token, islet p-PKCδ protein expression was also decreased in GK rats but we were not able to quantify it (Fig 3F). Finally, the ratio of p-PKCα to PKCα (p-PKCα/PKCα) in islets did not show any difference between the three groups of rats (Fig 3I). The islet p-PKCε/PKCε was reduced in GK compared with insulin-treated GK (p < 0.01) and Wistar rats (p < 0.05) (Fig 3J), which could be due to very low expression of p-PKCε (Fig 3G). Also, the islet p-PKCζ/PKCζ was increased in insulin-treated GK compared with Wistar (p < 0.05) and GK rats without insulin treatment (p < 0.05) (Fig 3K).

**Fig 4:**
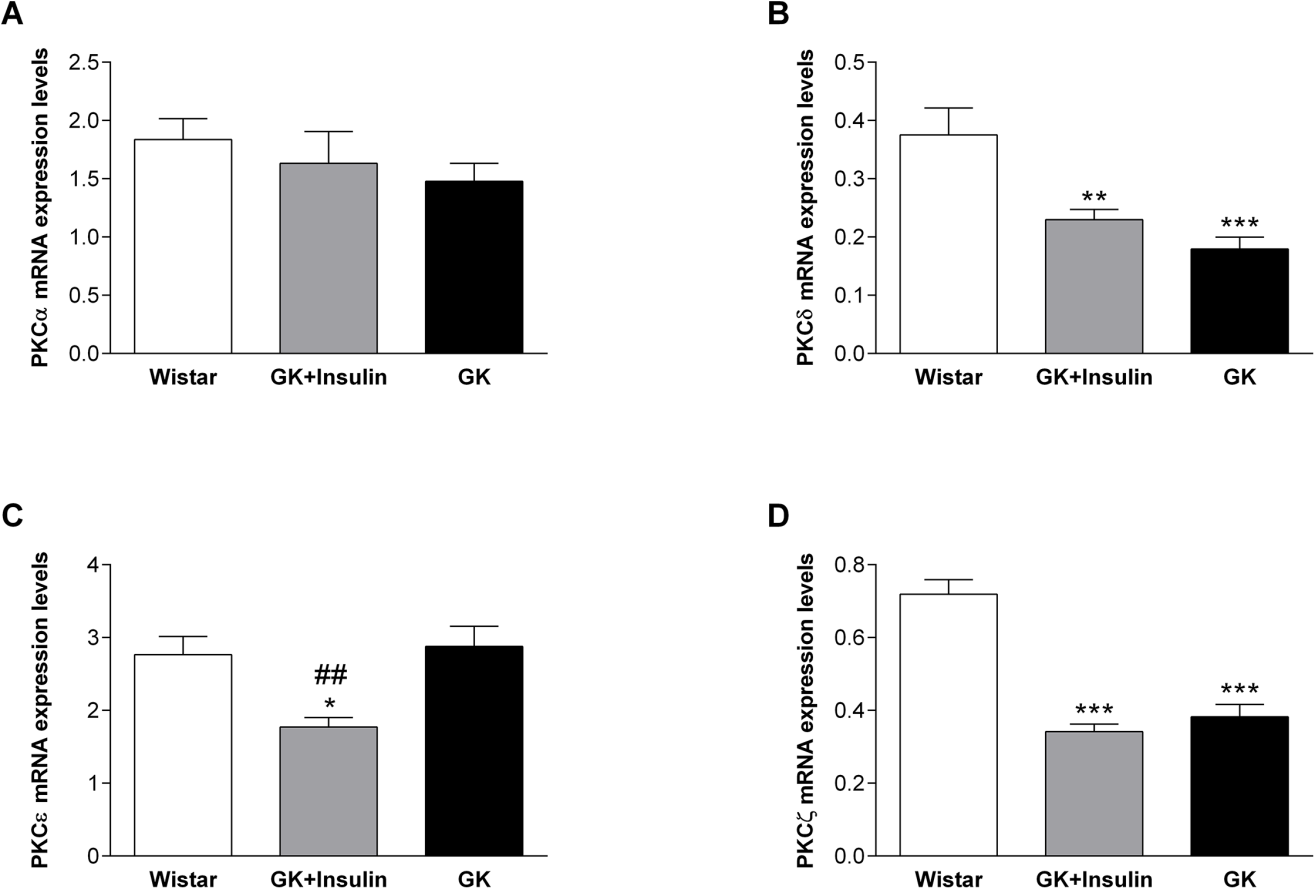
mRNA expressions of PKC isoforms in liver. mRNA expression levels of PKCα (A), PKCδ (B), PKCε (C), and PKCζ (D) in livers of Wistar, insulin-treated GK, and non-treated GK rats are shown. Data are means ± SE (n = 10). *p < 0.05, **p < 0.01, ***p < 0.001 vs. Wistar rats; ^##^p < 0.01 vs. GK rats.

**Fig 5:**
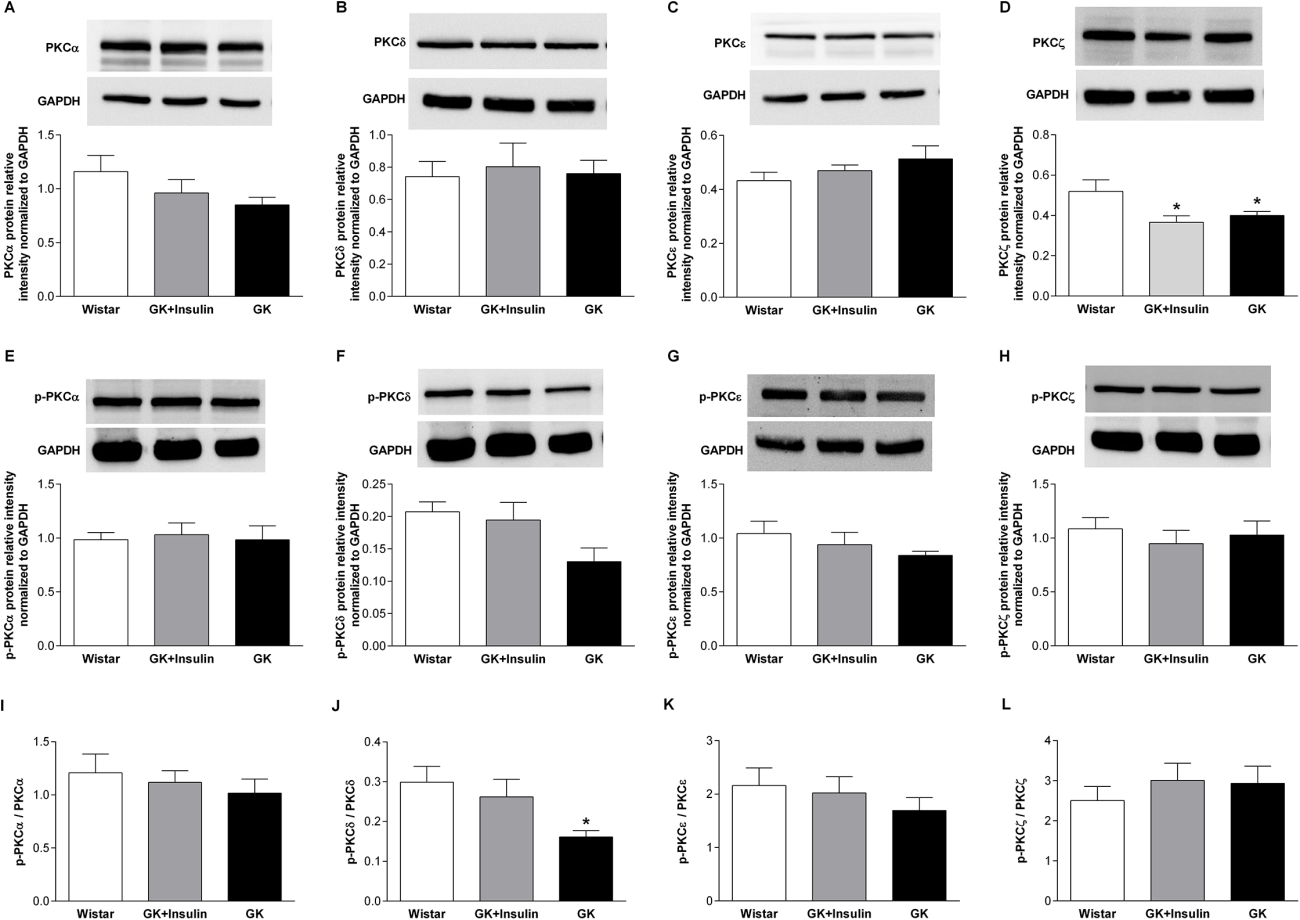
Protein expressions of PKC isoforms in liver. Representative immunoblots and densitometric analyses of PKCα (A), PKCδ (B), PKCε (C), PKCζ (D), p-PKCα (E), p-PKCδ (F), p-PKCε (G), and p-PKCζ (H) protein expressions in livers of Wistar, insulin-treated GK, and non-treated GK rats. Calculation of the ratio of phosphorylated protein to total protein allowed for assessing the sensitivity of proteins in livers from the three groups of rats (I-L). Data are means ± SE (n = 6). *p < 0.05 vs. Wistar rats.

In liver, PKCδ and PKCζ mRNA expressions were reduced in both GK (p < 0.001) and insulin-treated GK (p < 0.01 and p < 0.001, respectively) compared with Wistar rats (Fig 4B and 4D). Liver PKCζ protein expression was decreased in both GK rats with and without insulin treatment compared with Wistar rats (p < 0.05) (Fig 5D). Though hepatic PKCδ showed no difference at the protein level (Fig 5B and 5F), the p-PKCδ/PKCδ was reduced in GK compared with Wistar rats (p < 0.05) (Fig 5J). Although PKCε mRNA expression was under-expressed in liver of insulin-treated GK compared with non-treated GK (p < 0.01) and Wistar rats (p < 0.05) (Fig 4C), it showed no difference in expression at the protein level (Fig 5C, 5G and 5K). Also, PKCα in liver exhibited no difference between the three groups of rats either at the mRNA or protein levels (Fig 4A, and Fig 5A, 5E and 5I).

Table 1 summarizes mRNA and protein expression levels of PKC isoforms in pancreatic islets and liver of GK and insulin-treated GK compared to Wistar rats.

**Table 1:** Summary of mRNA and protein expression levels of PKC isoforms in pancreatic islets and liver of GK and insulin-treated GK compared to Wistar rats. ↓, decreased; ns, no significant change relative to Wistar rats; int., intermediate; 1°, primary defect; 2°, secondary to the diabetic state (hyperglycaemia).

PKC isoform	Islets mRNA	Islets protein	Liver mRNA	Liver protein
PKCα	↓ (int.)	↓ (2°)	ns	ns
PKCδ	ns	↓ (2°)	↓ (1°)	ns
PKCε	↓ (2°)	↓ (2°)	ns^a^	ns
PKCζ	↓ (int.)	↓ (1°)	↓ (1°)	↓ (1°)

^a^down-regulated in insulin-treated GK compared to GK and Wistar rats.

## Discussion

In the current study, we have confirmed and extended previous observations of expression levels of PKC isoforms in pancreatic islets and liver of GK rats, namely PKCα, PKCδ, PKCε, and PKCζ. Also, we have assessed their association with glucose homeostasis and whether the impaired expression of the targeted PKC isoforms is a primary defect or rather secondary to hyperglycaemia by normalizing plasma glucose levels in the GK rats through insulin treatment. In this context, however, it is important to keep in mind that insulin itself, directly or indirectly by modulating other factors than glucose, e.g. lipids, may also exert effects on the PKCs [12–14].

We have found that PKCα and PKCζ mRNA expressions in pancreatic islets were diminished in GK compared with Wistar rats, with intermediate expressions in the insulin-treated GK group. PKCα and p-PKCα protein expressions in islets were reduced in GK compared with insulin-treated GK and Wistar rats. PKCζ protein expression in islets was decreased in both GK and insulin-treated GK compared with Wistar rats, but p-PKCζ was reduced only in GK compared with insulin-treated GK and Wistar rats. Islet PKCε was lower in GK compared with insulin-treated GK and Wistar rats at both mRNA and protein levels. PKCδ and p-PKCδ protein expressions in islets were decreased in GK compared with insulin-treated GK and Wistar rats. In liver, PKCδ and PKCζ mRNA expressions were decreased in both GK and insulin-treated GK compared with Wistar rats. Hepatic PKCζ protein expression was diminished in GK rats with and without insulin treatment compared with Wistar rats. Although PKCδ showed no difference at the protein level, the p-PKCδ/PKCδ was reduced in liver of GK compared with Wistar rats. PKCε mRNA expression in liver was under-expressed in insulin-treated GK compared with non-treated GK and Wistar rats.

In the GK rat pancreas, immunohistochemical staining has proved diminished expression of at least four different PKC isoenzymes: PKCα, PKCε, PKCθ, and PKCζ [23]. PKCα is suggested to play a role in insulin granule recruitment for exocytosis and insulin secretion in response to glucose [23, 28–33], and its expression is found to be modulated by hyperglycaemia [23,34]. This is consistent with our finding in the present study in which PKCα was down-regulated in pancreatic islets of GK compared with Wistar rats, with intermediate expression in insulin-treated GK rats, suggesting a contribution of hyperglycaemia to the reduced levels in GK rat islets. Of note, GSIS was higher in islets from insulin-treated vs. non-treated GK rats, suggesting at least a partial restoration of insulin exocytosis.

PKCδ is reported to play a non-redundant role in GSIS from pancreatic β-cells [35]. Contradictorily, the overexpression of kinase-negative PKCδ in pancreatic β-cells protects mice from high-fat diet-induced glucose intolerance and β-cell dysfunction [36]. These seemingly opposing findings could be explained by the difference between the whole-body knockout versus the β-cell-specific inhibition of PKCδ performed in these two studies, respectively. In addition, the latter study investigated the effect of PKCδ in glucose homeostasis after high-fat feeding. Furthermore, Frangioudakis *et al*. have reported that deletion of PKCδ protects against high-fat diet-induced glucose intolerance and improves glucose tolerance in chow-fed mice [37]. Data from our present study showed that islet PKCδ mRNA expression was not affected in non-treated GK rats. Nevertheless, the PKCδ and p-PKCδ protein expression were impaired in the GK rat islets, but the insulin-treated GK group exhibited increased protein expression to levels comparable to those of Wistar rats. In liver, PKCδ is proposed to regulate hepatic insulin sensitivity and hepatosteatosis in mice and men [24]. Global or liver-specific inactivation of PKCδ results in improved glucose tolerance and insulin sensitivity. Moreover, liver-specific over-expression of PKCδ leads to hepatic insulin resistance. Also, PKCδ expression is enhanced in livers of obese and T2D obese subjects [24]. In the current study, liver mRNA expression of PKCδ was down-regulated in the GK rat, which is a non-obese diabetic model. While hepatic PKCδ and p-PKCδ protein expressions were not affected in GK rats, the p-PKCδ/PKCδ was decreased in GK compared with Wistar rats; and this goes well with the fact that insulin resistance is not the main, albeit contributing, pathophysiological feature in the diabetic state in GK rats [8,38].

PKCε has been identified to be implicated in the regulation of survival pathways via activation of Akt in many cell types [39]. PKCε protects islets and improves their survival during the phase of isolation, contributing to preserved islet cell mass [40]. Incidentally, deletion of PKCε, under conditions of lipid oversupply, improves GSIS and prevents glucose intolerance in mice [41]. In the present study, islet PKCε was down-regulated in GK rats at the mRNA and protein levels. In liver, PKCε mRNA, but not protein, was decreased in insulin-treated GK rats, suggesting insulin involvement in its control at least at the mRNA level. This finding is in concert with previous observations showing a crucial role of PKCε in mediating lipid-induced hepatic insulin resistance [18].

PKCζ, through mTOR activation, is essential for growth factor-induced pancreatic β-cell proliferation with a concomitant improvement in β-cell function [42–44]. In this context it is of interest that GK rats from the Paris colony display at fetal stage a reduction of the pancreatic β-cell mass, which is maintained in the adult animal and apparently predates the onset of diabetes (hyperglycaemia) at about three or four weeks of age [45]. In contrast, in the Stockholm colony, β-cell density and relative volume of islet endocrine cells were alike in two- to three-month-old GK rats and control Wistar rats [8]. In addition, GK pups from the Stockhom colony were already hyperglycaemic at the first week of age [5]. Although PKCζ protein expression, in comparison with Wistar rats, was decreased in islets of GK and insulin-treated GK rats in the current study, the latter group demonstrated considerable enhancement in phosphorylating PKCζ and the p-PKCζ/PKCζ was increased in insulin-treated GK rats compared with both non-treated GK and Wistar rats. The significance of this enhancement needs to be elucidated further. In liver cells, PKCζ mediates the PI3K effect on insulin internalization in a Rab5-dependent manner [46]. Given that defective insulin internalization and, consequently, hyperinsulinaemia may also cause secondary insulin resistance in animal models [47], PKCζ activation could be important for improving insulin sensitivity. Though hepatic PKCζ protein expression was decreased in both GK rats with and without insulin treatment compared with Wistar rats in the current study, the p-PKCζ and p-PKCζ/PKCζ were not different between the three groups of rats, and that could be explained by the fact that the GK rat is not a severely insulin-resistant model. Collectively, enhancing PKCζ activity could be useful in therapeutic strategies for the treatment of diabetes.

In GK rat skeletal muscle exposed to hyperglycemia *in vivo*, increased phosphorylation of PKCα, PKCδ and PKCζ indicated defective insulin signalling, and this was not corrected by acute normalization of glycemia [16]. Nevertheless, the long-term consequence of elevated PKC-phosphorylation/activity should be considered in diabetes with chronic hyperglycemia [16]. While in our study, insulin treatment of GK rats improved the phosphorylation of some PKC isoforms, the expression of PKCζ in pancreatic islets and liver still remained impaired.

A previous investigation pointed at the possibility that PKC activation may contribute to impaired glycogen synthase activation in GK rat skeletal muscle. Thus, hyperinsulinemia was associated with persistent membrane translocation and activation of PKCs, that in turn may lead to impaired glycogen synthesis and insulin resistance [15]. However, the hyperinsulinemia induced by insulin treatment in the GK rats did not impair PKC activation in liver and could be explained by inhibition of insulin resistance in this animal model. Hepatic insulin resistance is often associated with an increase in hepatic diacyl-glycerol (DAG), that leads to activation of PKCε [18]. In our investigation, the insulin treatment of GK rats did not affect hepatic PKCε activation, suggesting that the insulin treatment modulated PKCε activation in a way to avoid hepatic insulin resistance.

Although we have demonstrated effects of insulin treatment on expression of PKC isoforms in pancreatic islets and liver, we cannot exclude that insulin itself or insulin-dependent factors other than glucose could influence PKC isoform expression. For example, insulin suppresses free fatty acid (FFA) concentrations by inhibiting lipolysis in the adipose tissue, and FFA [48,49] and lipolysis [50–52] are known to activate PKC and thus could partly explain our results.

In conclusion, the present study in pancreatic islets of GK rats suggests defects in PKCα, PKCε, and p-PKCζ expressions secondary to hyperglycaemia, since the expression pattern was restored after insulin treatment. It is however possible that also insulin itself may exert effects on the PKC isoform expression. In liver, PKCδ and PKCζ mRNA expressions were primarily linked to hyperglycaemia. In addition, PKCε mRNA expression in liver could be under control of insulin. Moreover, the capacity of hepatic PKCδ to be phosphorylated is diminished in GK rats. Indeed, the insulin treatment has improved GSIS from pancreatic islets of GK rats, and supports the view that early improvement of glycemia is beneficial in the management of T2D.

## Supporting Information

S1 Fig Characteristics of diabetic GK and control Wistar rats. (PDF)

S2 Fig Islet mRNA expression of PKC isoforms. (PDF)

S3 Fig Islet protein expression of PKC isoforms. (PDF)

S4 Fig Liver mRNA expression of PKC isoforms. (PDF)

S5 Fig Liver protein expression of PKC isoforms. (PDF)
