# Correction: Expression of Protein Kinase C Isoforms in Pancreatic Islets and Liver of Male Goto-Kakizaki Rats, a Model of Type 2 Diabetes

**DOI:** 10.1371/journal.pone.0141292

**Published:** 2015-10-20

**Authors:** Mohammed Seed Ahmed, Julien Pelletier, Hannes Leumann, Harvest F. Gu, Claes-Göran Östenson

The first author’s name appears incorrectly in the citation. The correct citation is: Seed Ahmed M, Pelletier J, Leumann H, Gu HF, Östenson C-G (2015) Expression of Protein Kinase C Isoforms in Pancreatic Islets and Liver of Male Goto-Kakizaki Rats, a Model of Type 2 Diabetes. PLoS ONE 10(9): e0135781. doi:10.1371/journal.pone.0135781

## References

[1] Ahmed MS, Pelletier J, Leumann H, Gu HF, Östenson C-G (2015) Expression of Protein Kinase C Isoforms in Pancreatic Islets and Liver of Male Goto-Kakizaki Rats, a Model of Type 2 Diabetes. PLoS ONE 10(9): e0135781 doi:10.1371/journal.pone.0135781 26398746 10.1371/journal.pone.0135781 PMC4580567

