# Supplementary material for: Expression of Protein Kinase C Isoforms in Pancreatic Islets and Liver of Male Goto-Kakizaki Rats, a Model of Type 2 Diabetes
Source: PLoS One. 2015 Sep 23;10(9):e0135781. doi: 10.1371/journal.pone.0135781 (PMC4580567; doi:10.1371/journal.pone.0135781)
Supplement: S1 Fig — (PDF) [file pone.0135781.s001.pdf]

Body Weight (g)	Wistar	GK+Insulin	GK	Wistar D15	GK+Insulin D15	GK D15
	5,8	14,3	9,9	5,5	6	9,2
	6	10,2	18	7	6,2	19,8
	6,9	10,6	7,5	5,7	8,3	7,8
	6,2	13,3	6,4	6,1	5,6	6,1
	5,9	10	10,6	5	5,7	8,5
	6,8	6,4	15,9	5,3	5,5	20,6
	6,3	11,8	6,2	5,8	6,7	8
	5,5	8,1	8,8	5,8	3,4	8,7
	6,7	8	7,9	6	4,6	8,4
	6,6	8,3	6	6,3	3,9	6,9
	7	7,3	6	6	5	7,8
	6,5			5		
	6,5			5,2		
	6,8			6		

Serum Insulin (µU/ml)	GK	GK+Insulin	Wistar
	59	39	31
	55	117	25
	74	71	27
	64	72	30
	23	38	23
	31	57	23
	41	58	15
	53	54	20
	43	86	27
	42	112	28

Blood glucose (mmol/l)	Wistar	GK+Insulin	GK	Wistar D15	GK+Insulin D15	GK D15
	5,8	14,3	9,9	5,5	6	9,2
	6	10,2	18	7	6,2	19,8
	6,9	10,6	7,5	5,7	8,3	7,8
	6,2	13,3	6,4	6,1	5,6	6,1
	5,9	10	10,6	5	5,7	8,5
	6,8	6,4	15,9	5,3	5,5	20,6
	6,3	11,8	6,2	5,8	6,7	8
	5,5	8,1	8,8	5,8	3,4	8,7
	6,7	8	7,9	6	4,6	8,4
	6,6	8,3	6	6,3	3,9	6,9
	7	7,3	6	6	5	7,8
	6,5			5		
	6,5			5,2		
	6,8			6		

S1_fig.
