# Supplementary material for: Expression of Protein Kinase C Isoforms in Pancreatic Islets and Liver of Male Goto-Kakizaki Rats, a Model of Type 2 Diabetes
Source: PLoS One. 2015 Sep 23;10(9):e0135781. doi: 10.1371/journal.pone.0135781 (PMC4580567; doi:10.1371/journal.pone.0135781)
Supplement: S2 Fig — (PDF) [file pone.0135781.s002.pdf]

ISLETS	GK	GK+Insulin	Wistar
mRNA PKC-Alpha	0,328445	0,4755208	0,4810606
	0,2030413	0,354285	1
	0,2586207	0,4904217	0,3944837
	0,2803499	0,3558845	0,4875161
	0,274423	0,2762557	0,3132871
	0,4164151	0,3799646	0,183168
	0,2487715	0,30339	0,2302164
	0,1982111	0,3628122	0,3681714
	0,2316176	0,3020433	0,4505135
	0,2233268	0,2446603	
mRNA PKC-Delta	0,9788288	1,976618	2,252381
	1,409542	2,034745	2,75
	1,909926	2,988426	1,023268
	1,239249	0,9561278	0,738721
	1,457265	0,9721598	0,9335992
	1,988095	2,265652	0,9700041
	2,55873	1,395657	1,550586
	1,231537	1,273656	0,8663619
	2,271028	3,39565	0,6012594
	0,767795	2,008754	

ISLETS	GK	GK+Insulin	Wistar
mRNA PKC-Epsilon	1,228116	2,540353	2,9
	1,135903	1,66073	4,5
	0,9356872	1,946808	1,921181
	1,158432	1,936763	2,512086
	1,662745	1,6489	1,836394
	2,471781	2,195745	1,398181
	1,731971	1,705314	1,660345
	1,163034	1,926319	2,110579
	1,789967	3,478022	2,192474
	0,5097898	1,516656	
mRNA PKC-Zeta	1,886782	3,342921	5,757143
	1,131161	3,32118	4,5
	1,800793	1,486286	2,155711
	1,230159	1,963274	3,333936
	2,731624	2,001238	3,115948
	2,760173	3,596411	2,821429
	2,203532	1,781486	1,980903
	1,216627	1,521404	3,002959
	1,960382	3,309524	3,064972
	0,7869212	1,475582	

S2_fig.
