# Supplementary material for: Expression of Protein Kinase C Isoforms in Pancreatic Islets and Liver of Male Goto-Kakizaki Rats, a Model of Type 2 Diabetes
Source: PLoS One. 2015 Sep 23;10(9):e0135781. doi: 10.1371/journal.pone.0135781 (PMC4580567; doi:10.1371/journal.pone.0135781)
Supplement: S3 Fig — (PDF) [file pone.0135781.s003.pdf]

ISLETS	GK	GK+Insulin	Wistar
PKC-Alpha	0,4969586	0,8340981	0,7788108
	0,6129774	0,9374765	0,6456187
	0,4938452	0,8423856	0,5765146
	0,4559852	0,7481809	0,6863826
Phosphorylated PKC-Alpha	0,4223157	0,7026443	0,8780853
	0,5143377	0,9525915	0,6678087
	0,4541526	0,6325659	0,5855735
	0,4399467	0,6091071	0,5892638
Ratio p-PKC-Alpha/PKC-Alpha	0,849801	0,8424	1,127469
	0,839081	1,016123	1,03437
	0,919625	0,750922	1,015713
	0,964827	0,814117	0,858506

ISLETS	GK	GK+Insulin	Wistar
PKC-Epsilon	0,7053466	1,009962	1,095167
	0,5921097	0,8775189	0,9187295
	0,7088317	0,8408476	0,8160928
	0,7411501	0,8804455	0,801297
Phosphorylated PKC-Epsilon	0,1057632	0,223295	0,2199267
	0,1139235	0,2410471	0,268544
	0,11322736	0,2770063	0,14503
	0,08370057	0,3888934	0,2668917
Ratio p-PKC-Epsilon/PKC-Epsilon	0,149945	0,221093	0,200816
	0,192403	0,274692	0,292299
	0,159738	0,329437	0,177713
	0,112933	0,441701	0,333075

ISLETS	GK	GK+Insulin	Wistar
PKC-Delta	0,05221694	0,4445191	0,3376693
	0,04852548	0,3502993	0,4824271
	0,026897	0,5864576	0,1795419
	0,02308936	0,2874383	0,1832878
Phosphorylated PKC-Delta	-	-	-
	-	-	-
	-	-	-
	-	-	-
Ratio p-PKC-Delta/PKC-Delta	-	-	-
	-	-	-
	-	-	-
	-	-	-

ISLETS	GK	GK+Insulin	Wistar
PKC-Zeta	0,14906	0,174448	0,289769
	0,1612355	0,193639	0,3849238
	0,2869013	0,2469482	0,3397637
	0,2402505	0,2196337	0,5054368
Phosphorylated PKC-Zeta	0,124493	0,4325598	0,2368122
	0,1931639	0,275125	0,344621
	0,1747647	0,255522	0,2321489
	0,1247763	0,3754075	0,3537493
Ratio p-PKC-Zeta/PKC-Zeta	0,835187	2,479579	0,817245
	1,198023	1,420814	0,895297
	0,957698	1,034719	0,683266
	0,519359	1,709244	0,699888

S3_fig
