# Supplementary material for: Expression of Protein Kinase C Isoforms in Pancreatic Islets and Liver of Male Goto-Kakizaki Rats, a Model of Type 2 Diabetes
Source: PLoS One. 2015 Sep 23;10(9):e0135781. doi: 10.1371/journal.pone.0135781 (PMC4580567; doi:10.1371/journal.pone.0135781)
Supplement: S4 Fig — (PDF) [file pone.0135781.s004.pdf]

LIVER	GK	GK+Insulin	Wistar
mRNA PKC-Alpha	1,290073	1,148602	1,086961
	1,233261	2,001739	1,756597
	1,363145	2,06497	1,464129
	1,201025	1,774023	1,915738
	1,014616	0,9072092	1,595195
	1,248845	1,716013	2,418869
	1,083814	0,6279239	2,498146
	2,63173	3,965293	0,7621544
	1,449149	1,371474	2,137178
	1,4211	1,440068	2,499753
	2,312141	0,9339659	1,844211
			1,465069
			3,195714
			1,085124
mRNA PKC-Delta	0,2071492	0,2797601	0,4206156
	0,140285	0,2390396	0,6896214
	0,1152272	0,1788671	0,805145
	0,1667898	0,2346539	0,2014633
	0,1575871	0,2005766	0,3370069
	0,3694668	0,3147663	0,3821668
	0,1412316	0,3367592	0,2953528
	0,1507933	0,1587983	0,2103344
	0,1841535	0,1959499	0,3477915
	0,1609583	0,1934134	0,2935622
	0,1806248	0,1965703	0,2357209
			0,3179541
			0,2831843
			0,4303791

LIVER	GK	GK+Insulin	Wistar
mRNA PKC-Epsilon	3,894472	1,522156	1,923175
	2,568159	1,953479	2,051603
	2,42211	2,626188	1,932093
	3,589929	1,763995	1,602409
	2,480357	1,218015	1,570588
	4,782648	1,467607	2,00703
	2,782853	1,757243	4,313583
	3,291752	2,330709	3,218457
	2,053979	1,648254	3,843635
	1,865028	1,283233	3,376539
	1,941061	1,920179	3,367558
			2,310189
			3,712205
			3,480604
mRNA PKC-Zeta	0,4923832	0,2371628	0,7571625
	0,485994	0,4380684	1,043675
	0,3165651	0,3296918	0,6957746
	0,2785545	0,3219248	0,5378554
	0,2896171	0,3007733	0,7237991
	0,6397521	0,3517016	0,7728978
	0,331338	0,3239994	0,5206391
	0,3989057	0,45108	0,7133222
	0,3076652	0,426023	0,9813834
	0,3226505	0,2776171	0,6897194
	0,339931	0,2993476	0,6887305
			0,7427688
			0,6621455
			0,5358898

S4_fig.
