# Supplementary material for: Expression of Protein Kinase C Isoforms in Pancreatic Islets and Liver of Male Goto-Kakizaki Rats, a Model of Type 2 Diabetes
Source: PLoS One. 2015 Sep 23;10(9):e0135781. doi: 10.1371/journal.pone.0135781 (PMC4580567; doi:10.1371/journal.pone.0135781)
Supplement: S5 Fig — (PDF) [file pone.0135781.s005.pdf]

LIVER	GK	GK+Insulin	Wistar
PKC-Alpha	0,9599607	0,9499704	0,9767252
	0,9632044	1,026988	1,333187
	0,833939	1,426788	1,384791
	0,64763	0,5165499	0,6252686
	0,9710014	1,049442	1,655027
	0,7113382	0,7961578	0,9790962
Phosphorylated PKC-Alpha	0,952164	0,661705	1,126803
	1,301895	1,170162	1,102502
	1,107637	1,087145	0,768943
	0,749879	1,345469	1,036147
	1,138603	1,165151	1,083036
	0,619855	0,75141	0,789405
Ratio p-PKC-Alpha/PKC-Alpha	0,991878105	0,696553282	1,153654068
	1,351629	1,139411561	0,82696726
	1,328199065	0,761952722	0,555277295
	1,157881815	2,604722216	1,657123035
	1,17260696	1,110257642	0,654391741
	0,87139282	0,943795313	0,806258874

LIVER	GK	GK+Insulin	Wistar
PKC-Delta	0,5522804	0,6491481	0,667532
	0,6550392	0,6400918	0,799521
	0,6698158	0,6305004	0,7456512
	1,125655	1,524108	1,08646
	0,7641127	0,7725423	0,7805088
	0,8016371	0,6032478	0,3752744
Phosphorylated PKC-Delta	0,056177	0,123848	0,177076
	0,090472	0,298917	0,180981
	0,11639	0,149011	0,203503
	0,18574	0,235992	0,277054
	0,127159	0,211125	0,220188
	0,177186	0,149487	0,18474
Ratio p-PKC-Delta/PKC-Delta	0,101719	0,190786	0,26527
	0,138117	0,466991	0,226361
	0,173764	0,236338	0,272919
	0,165006	0,154839	0,255006
	0,166415	0,273286	0,282108
	0,22103	0,247804	0,492279

S5_fig.

LIVER	GK	GK+Insulin	Wistar
**PKC-Epsilon**	0,6053609	0,4996566	0,4883186
	0,6311706	0,5036214	0,4801016
	0,3912952	0,4022116	0,489721
	0,3803953	0,5350199	0,4652481
	0,6224246	0,4492286	0,3596198
	0,4450716	0,4257801	0,349657
**Phosphorylated PKC-Epsilon**	0,7311746	0,7147362	0,7088225
	0,7766124	0,7518969	0,7726724
	0,8990208	0,8470514	0,9875568
	0,8494669	1,048828	1,149859
	0,9373476	1,079899	1,173479
	0,867072	1,329374	1,161071
**Ratio p-PKC-Epsilon/PKC-Epsilon**	1,207833	1,430455	1,451557
	1,230432	1,49298	1,609394
	2,297551	2,105985	2,01657
	2,233116	1,960353	2,471496
	1,505962	2,403896	3,26311
	1,948162	3,122208	3,320599

LIVER	GK	GK+Insulin	Wistar
**PKC-Zeta**	0,369947	0,3583014	0,4592628
	0,4501382	0,3718895	0,3805318
	0,3355307	0,4069779	0,746435
	0,4007806	0,4779709	0,4752202
	0,4550435	0,3337491	0,6343768
	0,3879765	0,250776	0,4235767
**Phosphorylated PKC-Zeta**	1,164685	1,106069	1,503187
	0,7410826	0,6137695	1,169067
	1,60889	1,457819	0,8465306
	0,9489232	0,727942	0,8021134
	0,9260215	*0,8937464*	1,075318
	0,7716431	0,8889002	*1,117981*
**Ratio p-PKC-Zeta/PKC-Zeta**	3,148248	3,086979	3,273043
	1,646345	1,650408	3,072193
	4,79506	3,582059	1,134098
	2,367687	1,522983	1,687877
	2,035017	2,677899	1,695077
	1,98889	3,544598	2,639382

S5_fig.
